# The haematopoietic GTPase RhoH modulates IL3 signalling through regulation of STAT activity and IL3 receptor expression

**DOI:** 10.1186/1476-4598-9-225

**Published:** 2010-08-25

**Authors:** Mehtap S Gündogdu, He Liu, Daniela Metzdorf, Dagmar Hildebrand, Michael Aigner, Klaus Aktories, Klaus Heeg, Katharina F Kubatzky

**Affiliations:** 1 Department für Infektiologie, Medizinische Mikrobiologie und Hygiene, Ruprecht-Karls-Universität, Im Neuenheimer Feld 324, D-69120 Heidelberg, Germany; 2 Institut für Experimentelle und Klinische Pharmakologie and Toxikologie, Albert-Ludwigs-Universität, Albertstraße 25, D-79104 Freiburg, Germany; 3 Department of Pharmacology, University of Bern, Friedbühlstr. 49, CH-3010 Bern, Switzerland; 4 Medizinische Klinik 5, Hämatologie und Internistische Onkologie, Universitätsklinikum Erlangen, Glückstr. 4a, D-91054 Erlangen, Germany

## Abstract

**Background:**

RhoH is a constitutively active member of the family of Rho GTPases. Its expression is restricted to the haematopoietic lineage, where it serves as a positive regulator for T cell selection and mast cell function and as a negative regulator for growth-related functions in other lineages. Here, we examined the activation of signal transducer and activator of transcription (STAT) proteins in response to stimulation with interleukin 3 (IL3).

**Results:**

Using the murine IL3-dependent cell line BaF3 we investigated the influence of RhoH protein expression levels on IL3-mediated cellular responses. RhoH overexpressing cells showed lower sensitivity to IL3 and decreased STAT5 activation. SiRNA-mediated repression of *RhoH *gene expression led to an increase in proliferation and STAT5 activity which correlated with an increased number of IL3 receptor α chain molecules, also known as CD123, expressed at the cell surface. Interestingly, these findings could be reproduced using human THP-1 cells as a model system for acute myeloid leukaemia, where low RhoH levels are known to be an unfavourable prognostic marker. Overexpression of RhoH on the other hand caused an induction of STAT1 activity and western blot analysis revealed that activated STAT1 is phosphorylated on Tyr701. STAT1 is known to induce apoptosis or cell cycle arrest and we detected an upregulation of cyclin-dependent kinase inhibitors (CDKI) *p21^Cip1 ^*and *p27^Kip1 ^*in RhoH overexpressing BaF3 cells.

**Conclusions:**

We propose that RhoH functions as a negative regulator for IL3-induced signals through modulation of the JAK-STAT pathway. High levels of RhoH allow the IL3-dependent activation of STAT1 causing decreased proliferation through upregulation of *p21^Cip1 ^*and *p27^Kip1^*. Low RhoH levels on the other hand led to an upregulation of IL3-dependent cell growth, STAT5 activity and an increase of CD123 surface expression, linking RhoH to a CD123/STAT5 phenotype that has been described in AML patients.

## Background

Rho GTPases belong to the superfamily of Ras GTPases [1] and function as molecular switches that control and integrate signal transduction pathways by linking receptor-derived signals to downstream signalling proteins [2-4]. The Rho subfamily of GTPases consists of 20 proteins, but only two members, Rac2 and RhoH, are specifically expressed in haematopoietic cells [5,6]. RhoH is a GTPase deficient protein [7,8] and its activity is presumably modulated through transcriptional regulation [7]. Recently it was found that RhoH activity can also be regulated by tyrosine phosphorylation of its non-canonical immune receptor tyrosine activation motif (ITAM) [9]. The protein was first discovered as a fusion transcript with the transcriptional repressor LAZ3/BCL6 in Non Hodgkin lymphoma cells [5]. In a number of B cell malignancies, RhoH is mutated with high frequency through somatic hypermutation [10,11]. In Hairy Cell Leukaemia (HCL) and Acute Myeloid Leukaemia (AML), RhoH was found to be underexpressed at the protein level [12,13]. The function of RhoH has been investigated in various haematopoietic cells and RhoH is thought to mainly act as a negative regulator for processes such as proliferation, survival, migration and engraftment of haematopoietic progenitor cells [14]. This is presumably due to the negative regulatory role RhoH has on Rac1 [7,13,15], although the exact mechanism remains to be elucidated. RhoH null mice showed impaired T cell differentiation due to defective T cell receptor signalling [9,16]. However, other functions of RhoH have now become known that had not been obvious from the knock-out animals [17-19]. In mast cells, for example, RhoH positively regulates signalling through the FcεR [18]. In neutrophils from patients suffering from chronic obstructive pulmonary disease [19] or cystic fibrosis [17], a GM-CSF-dependent upregulation of *RhoH *had been found. These data were corroborated using RhoH-deficient mice, showing that RhoH negatively regulates leukotriene production.

Here, we demonstrate that RhoH regulates interleukin 3 (IL3)-induced signalling through modulation of the activity of signal transducer and activator of transcription (STAT) proteins. Important functions of IL3 are the regulation of growth and early differentiation of haematopoietic progenitors [20] as well as the control of the terminal differentiation of basophils, mast cells and dendritic cells [21,22]. Recent publications suggest a strong link between RhoH expression levels and B cell malignancies [12,13]. We therefore used IL3-dependent BaF3 cells, a murine proB cell line, as a model system. These cells were shown to express comparatively low levels of RhoH [7]. We show that overexpression of RhoH decreases IL3-induced proliferation and the activity of STAT5. The surface expression level of the IL3 receptor α-chain (CD123) is inversely correlated to the expression levels of RhoH. In RhoH-deficient cells, the STAT5-dependent gene *interferon regulatory factor-1 *(*IRF-1*) is upregulated, eventually leading to an upregulation of CD123. Interestingly, only BaF3 cells that overexpress RhoH are able to activate STAT1 after stimulation with IL3. This correlates with an upregulation of the STAT1-dependent cell cycle inhibitors *p21^Cip1 ^*and *p27^Kip1^*. Thus, our findings link the regulatory function of RhoH on proliferation to an interaction with the JAK-STAT signalling pathway.

## Results

### RhoH regulates IL3-dependent cell proliferation

**Figure 1:**
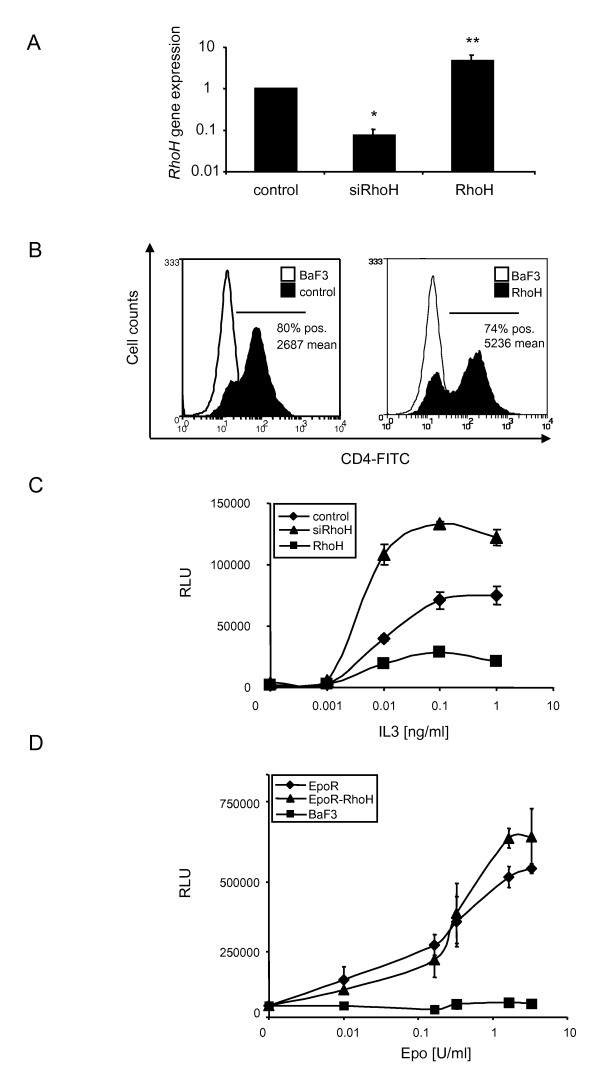
**RhoH regulates IL3-induced proliferation**. (A) *RhoH *mRNA expression levels in control cells, RhoH or siRhoH-transduced BaF3 cells were detected by quantitative real-time PCR using *RhoH *specific primers and *GAPDH *as a reference gene. Statistical significance was analysed using the 's t-test (mean ± SD, n = 2 and *P ≤ 0.05; **P ≤ 0.01). (B) FACS analysis of control cells transduced with the empty vector (pMX-IRES-CD4-Puro) or RhoH (pMX-IRES-CD4-Puro RhoH) -transduced BaF3 cells for human CD4 expression after puromycin selection. Parental BaF3 cells were used as a negative control. (C) Dose response curve of control cells, RhoH and siRhoH BaF3 cells cultivated with increasing concentrations of IL3. Viability was determined by quantification of cellular ATP in a Cell Titer Glo assay. Results shown are from one representative experiment performed in triplicates ± SD. (D) Dose response curve of BaF3 cells, BaF3-EpoR cells and BaF3-EpoR cells overexpressing RhoH cultivated with increasing concentrations of Epo. Viability was determined by quantification of cellular ATP in a Cell Titer Glo assay. Results shown are from one representative experiment performed in triplicates ± SD.

In order to study the effects of RhoH on IL3-mediated signals, we used the IL3-dependent, murine proB cell line BaF3 to generate cell lines where the *RhoH *gene was silenced (siRhoH) using the retroviral vector pSilencer Retro 5.1 U6 (Ambion, Austin, USA) or that were retrovirally transduced with RhoH to overexpress the protein (RhoH). As a control, parental BaF3 cells were transduced with the empty vector (pMX-IRES-CD4-Puro). Infected cells were selected with puromycin and the resulting stable cell lines were tested for RhoH expression by quantitative real-time PCR using *GAPDH *as a reference gene. Figure 1 A shows that in siRhoH cells the expression was decreased to app. 10% compared to control cells, while the RhoH cells showed a five-fold overexpression of the gene. To corroborate successful retroviral transduction, we performed FACS analysis and compared CD4 expression levels. It was shown previously that the level of expression of the genes up- and downstream of the IRES sequence are highly correlated in stably infected target cells [23]. Figure 1B shows that control cells and RhoH-transduced cells were app. 80% positive for CD4 expression and expressed the vector at comparable levels. Parental cells that do not express CD4 were used as a negative control.

To investigate whether the overexpression of RhoH leads to changes in IL3 receptor-mediated signalling events, proliferative responses were tested using control cells and BaF3 cells overexpressing RhoH (Figure 1C). Cells were incubated with IL3 concentrations ranging from 0.001 to 1 ng/ml and factor-dependent growth was determined after 48 hours through measurement of cellular ATP (Cell Titer Glo, Promega). In RhoH cells, the ability to proliferate in response to IL3 was reduced to app. 30%. On the other hand, siRhoH cells proliferated better in response to IL3 achieving a growth rate of 160% compared to cells transduced with the empty vector. Thus, the expression level of RhoH regulates the ability of BaF3 cells to proliferate in response to IL3. To clarify whether these findings were specific for IL3, we repeated the experiment with BaF3 cells transduced with erythropoietin receptor (EpoR). EpoR cells, EpoR-RhoH cells and parental BaF3 cells were cultivated at Epo concentrations between 0.01 and 6.5 U/ml and cell viability was again determined after 48 h (Figure 1D). Interestingly, no differences in Epo-induced growth could be detected between EpoR and EpoR-RhoH cells. Parental BaF3 cells were not able to grow, as expected, since they do not express the EpoR. We therefore conclude that RhoH specifically regulates IL3-induced proliferation.

### RhoH modulates IL3-induced STAT activation

**Figure 2:**
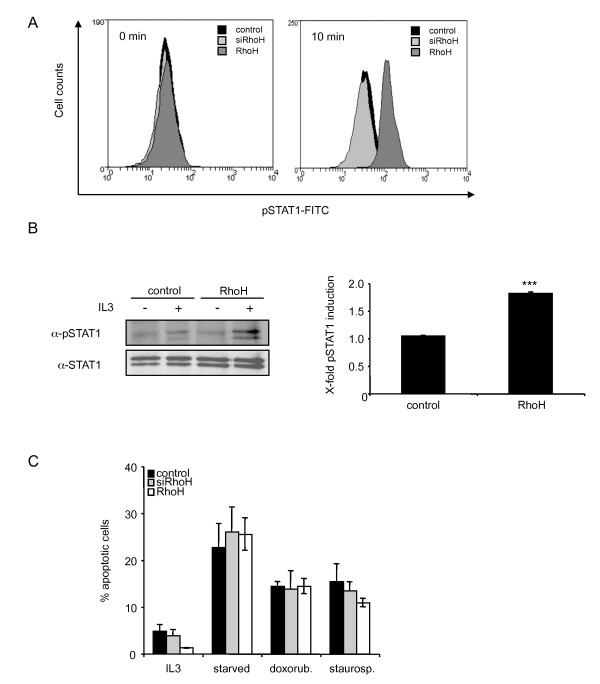
**RhoH expression triggers IL3-dependent STAT1 activation**. (A) Control cells, siRhoH and RhoH-transduced BaF3 cells were depleted of cytokine and FBS for 3 h prior to stimulation with IL3. Phosphorylated STAT1 levels were measured with intracellular FACS staining using a FITC-labelled pTyr-STAT1 specific antibody. One representative result of three independent experiments is shown. (B) Control and RhoH cells were starved for 3 hours prior to stimulation with 50 ng/ml IL3. Cells were lysed, STAT1 was immunoprecipitated and tyrosine phosphorylated STAT1 and total STAT1 were detected by enhanced chemiluminescence. Quantification of pSTAT1 levels was done after normalisation to STAT1 levels in cells transduced with the empty vector and is presented as induction of phosphorylation compared to the corresponding unstimulated sample. Statistical significance was analysed using the 's t-test (mean ± SD, n = 3; ***P ≤ 0.005). (D) Apoptosis was induced in control cells, siRhoH or RhoH-transduced cells by cultivation in the absence of IL3 (overnight), treatment with 1 μM staurosporine (3 h) or with 1 μM doxorubicine (12 h) prior to Annexin V/PI staining and FACS analysis (mean ± SD, n = 3).

Next, we investigated if the changes in cell proliferation were related to changes in the transduction of IL3-induced signals. STAT proteins are cytokine inducible transcription factors that act as regulators of proliferation and apoptosis. It was shown that overexpression of RhoH leads to a decrease in proliferation in murine haematopoietic progenitor cells that could be explained by an increased number of apoptotic cells [14]. In these studies, no signalling cascade was identified that could be responsible for a proapoptotic function of RhoH. Thus we examined whether the activity of STAT1 which is known to activate proapoptotic pathways, is modulated by the expression level of RhoH. We therefore stimulated control cells, siRhoH and RhoH cells for 10 min with 50 ng/ml IL3 and measured the phosphorylation status by intracellular FACS analysis (Figure 2A). The data show that there is no significant tyrosine phosphorylation detectable in vector transduced BaF3 or siRhoH cells in the presence or absence of IL3. In RhoH overexpressing cells, however, stimulation with IL3 induces an increase in STAT1 tyrosine phosphorylation. This finding was corroborated by performing a STAT1 immunoprecipitation and subsequent western blot analysis using a phospho-specific antibody (Figure 2B, left panel). To assess the total levels of STAT1 the blot was reprobed using a p84/p91 STAT1 antibody. The signal intensities were quantified and normalised to STAT1 expression levels of control cells. The resulting quantification shows that the phosphorylation levels of RhoH cells for STAT1 are app. two-fold higher compared to the control where no significant induction was detected (Figure 2B, right panel).

Since STAT1 is known to mainly transduce apoptotic or cycle arrest-inducing signals, we investigated whether we could observe increased apoptosis in RhoH cells. Apoptosis was induced in control cells, RhoH cells or siRhoH cells through withdrawal of cytokine or treatment with apoptosis inducing agents such as doxorubicin or staurosporine. Apoptosis was then measured by FACS analysis and subsequent quantification of Annexin-V positive cells. However, no difference in the susceptibility of RhoH cells was found compared to control cells (Figure 2C).

### Overexpression of RhoH leads to the upregulation of p21*^Cip1 ^*and p27*^Kip1^*

**Figure 3:**
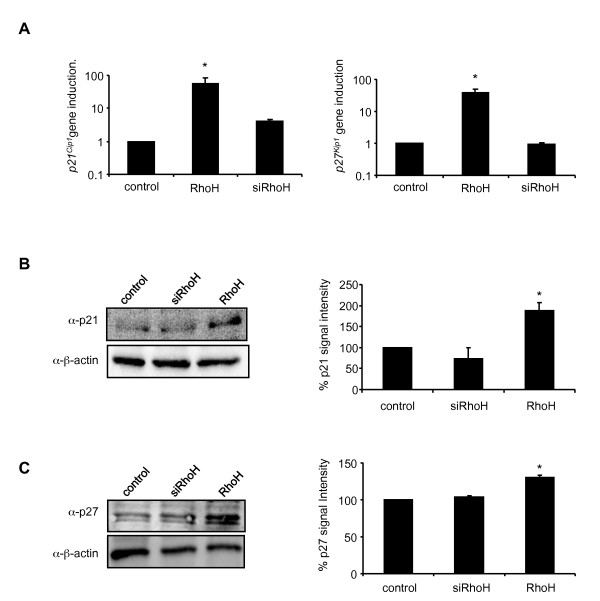
**RhoH upregulates p21*^Cip1 ^*and p27*^Kip1 ^*expression**. (A) Quantification of *p21^Cip1 ^*and *p27^Kip1 ^*mRNA expression levels by quantitative SYBR-green PCR in control cells, RhoH and siRhoH-transduced cells. Data were normalised to mRNA levels detected in control cells compared to *GAPDH*. Statistical significance was analysed using the 's t-test (mean ± SD, n = 3; *P ≤ 0.05). (B, C) Whole cell lysates of control cells, siRhoH and RhoH expressing cells were separated by 4-20% gradient SDS-PAGE and protein expression was detected by antibodies against p21*^Cip1 ^*and p27*^Kip1^*. Protein levels were normalised to β-actin expression (lower panel). Quantification and statistical significance were performed using the 's t-test (mean ± SD, n = 2; *P ≤ 0.05).

We therefore reasoned that RhoH may play a role in regulation of cell cycle progression, rather than apoptosis. The activities of cell cycle regulating cyclin dependent kinases (CDKs) are negatively controlled by the WAF/CIP family of CDK inhibitors. We examined the expression levels of the cyclin-dependent kinase inhibitors *p21^Cip1 ^*which is known to be a STAT1-induced gene [24] and *p27^Kip1 ^*of the same family by quantitative real-time PCR using *GAPDH *as a reference gene. Total RNA was prepared from control cells and RhoH cells and the gene expression of *p21^Cip1 ^*and *p27^Kip1 ^*were determined (Figure 3A). RhoH cells showed a 55 and 40-fold increase in the expression of *p21^Cip1 ^*and *p27^Kip1^*, respectively, compared to control cells. There were no significant changes in the expression of *p21^Cip1 ^*and *p27^Kip1 ^*detectable in siRhoH cells compared to the control. This increased expression was also found in IL3-treated bone marrow cells from wildtype compared to RhoH deficient mice (data not shown). To corroborate this finding also on the protein level, we prepared whole cell lysates from all three cell lines and subjected them to western blot analysis using p21*^Cip1 ^*and p27*^Kip1 ^*specific antibodies and β-actin as a loading control (Figure 3B and 3C). Again, we found p21*^Cip1 ^*and p27*^Kip1 ^*to be upregulated in cells overexpressing RhoH. The resulting quantification of the blots shows a statistically significant two-fold upregulation of p21*^Cip1^*expression. The detected p27*^Kip1 ^*expression is less prominent (1.3 fold) but reproducible. We therefore propose that the expression of RhoH modulates IL3-induced proliferation through upregulation of p21^Cip1 ^and p27^Kip1^expression and we suggest that this is a STAT1-dependent event.

### Underexpression of RhoH enhances STAT5 activity

**Figure 4:**
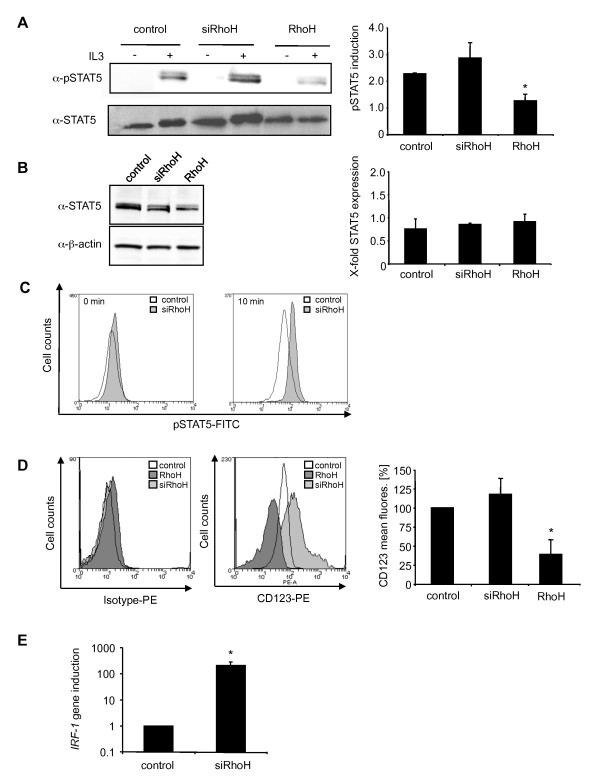
**Low RhoH expression levels enhance STAT5 activity**. (A) Control cells, siRhoH and RhoH expressing cells were starved for 3 h in the absence of cytokine and FBS before stimulation with 50 ng/ml IL3. Cells were lysed and STAT5 was immunoprecipitated. Tyrosine phosphorylated STAT5 and total STAT5 were detected by enhanced chemiluminescence using specific antibodies. Quantification of pSTAT5 levels was done after normalisation to total STAT5 levels in control cells and is presented as induction of phosphorylation compared to the corresponding unstimulated sample. Statistical significance was analysed using the 's t-test (mean ± SD, n = 2; *P ≤ 0.05). (B) To verify equal expression levels of STAT5 protein in all cell lines, STAT5 was detected from whole cell lysates and quantified after normalisation to β-actin levels of control cells (mean ± SD, n = 3). (C) pSTAT5 levels were measured by intracellular FACS staining at 0 and 10 min of stimulation of previously starved cells with 50 ng/ml IL3. Representative graphs from one out of three independently performed experiments are shown. (D) CD123 surface expression of control cells, siRhoH and RhoH cells determined by FACS analysis using a CD123-PE antibody. The left panel shows a typical example and the right panel summarises the data of the mean fluorescence signal and the statistical analysis (mean ± SD, n = 3, *P ≤ 0.05). (E) Quantification of *IRF-1 *mRNA expression levels of control cells and siRhoH-transduced cells was done by quantitative real-time PCR using *GAPDH *as a reference gene. Data were normalised to the expression in control cells and are presented as fold induction of the *IRF-1 *gene compared to control cells (mean ± SD, n = 3; *P ≤ 0.05).

It was recently shown that reduced RhoH levels can be connected to cancer and protection from apoptosis [10,12,13]. STAT5 is the major STAT protein activated by IL3 [25,26] and is described to induce anti-apoptotic genes and cell proliferation. Consequently, dominant-negative STAT5 leads to partial inhibition of IL3-induced proliferation [27]. We therefore examined the ability of RhoH or siRhoH cells to activate STAT5 after IL3 stimulation. Equal cell numbers of previously IL3-depleted BaF3 cells were stimulated with 50 ng/ml IL3 and STAT5 was precipitated from the resulting lysates. Western blot analysis (Figure 4A, left panel) and quantification of pSTAT5 levels (Figure 4A, right panel) that was corrected for the level of total STAT5 showed a strong reduction of STAT5 tyrosine phosphorylation compared to control cells (50%). This again corroborated our finding that proliferation in response to IL3 is decreased in RhoH overexpressing cells. Reduction of RhoH expression in siRhoH cells led to a small increase (1.3-fold) in STAT5 tyrosine phosphorylation compared to control cells that showed higher variations between independent experiments. A possible explanation for this observation is that parental BaF3 cells have already comparatively low *RhoH *levels so that a further decrease causes less prominent effects. As the amount of precipitated total STAT5 showed some variation when same cell numbers were used (1x10^7^/IP), we additionally quantified STAT5 expression levels comparing different pools of the transduced cell lines and using 20 μg of each lysate (Figure 4B) and did not find any difference (Figure 4B, right panel). Increased phosphorylation of STAT5 in siRhoH cells was confirmed using intracellular staining by FACS analysis using a FITC-labelled pSTAT5 antibody (Figure 4C).

### IL3 Receptor α-chain expression is negatively regulated by RhoH

The enhanced activation of STAT5 in cells that expressed low levels of RhoH could potentially be caused by more efficient downstream signalling events or by an increase in the expression of the ligand binding cell surface receptor, IL3Rα (CD123). We therefore determined the surface localisation of CD123 by FACS analysis using a PE-labelled CD123 antibody. Figure 4D shows that siRhoH cells express app. 25% more CD123 than control cells, while RhoH overexpressing cells show a decrease of CD123 expression of app. 50% as determined from three independent experiments.

Interestingly, it is known that a large number of AML patients show elevated expression of CD123 and hyperactivation of STAT5, which protects these cells from apoptosis [28]. It was found previously that the transcription factor interferon regulatory factor-1 (IRF-1) is highly overexpressed in AML eventually leading to the upregulation of the IRF-1-dependent gene *CD123 *[29]. Since IRF-1 expression can be induced by STAT5 [30], we checked whether we could detect an upregulation of *IRF-1 *in siRhoH cells. Indeed, *IRF-1 *expression was app. 200 fold higher in siRhoH cells compared to control cells (Figure 4E).

### The acute monocytic leukaemia cell line THP-1 displays an siRhoH phenotype

**Figure 5:**
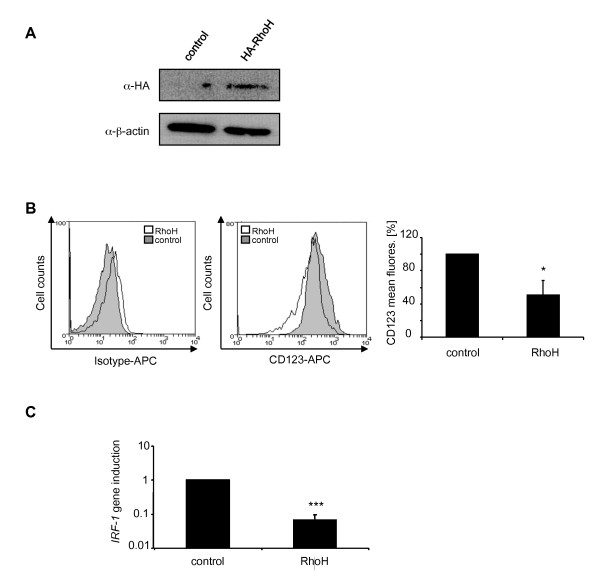
**The AML cell line THP-1 displays an siRhoH phenotype**. (A) THP-1 cells were either transfected with HA-RhoH or the empty vector, lysed and separated by SDS-PAGE. Detection of HA-RhoH was performed with an HA-specific antibody and enhanced chemiluminescence. As a loading control, the expression of β-actin was detected. (B) THP-1 cells transfected with HA-RhoH or the empty vector were analysed for CD123 expression by FACS using a CD123-APC antibody. Data are presented as percentage of the mean fluorescence signal for CD123 (mean ± SD, n = 3, *P ≤ 0.05). (C) *IRF-1 *mRNA expression is decreased in RhoH over-expressing THP-1 cells. Quantification of *IRF-1 *mRNA expression levels by quantitative SYBR-green PCR in control cells and RhoH-transfected THP-1 cells using human *IRF-1 *specific primers and *GAPDH *as a reference gene. Data were normalised to the expression in control cells and are presented as fold induction (mean ± SD, n = 3; ***P ≤ 0.005).

Low RhoH expression levels in samples from AML patients represent an unfavourable prognostic factor regarding patient survival. It was speculated that this might be connected to an increased resistance of these cells to apoptosis during chemotherapy through increased Rac1 activity [13]. To investigate whether our findings on the regulation of the anti-apoptotic factor STAT5 through low RhoH expression levels might have consequences in this scenario, we used THP-1 cells as a model system. THP-1 cells are derived from a patient with acute monocytic leukaemia. THP-1 cells correspond to the M5 phenotype in the French-American-British classification system of AMLs which were shown to have low levels of RhoH [13]. As a control, we used THP-1 cells that had been transiently transfected with human RhoH cDNA. Transfection efficiency was analysed by western blot using an HA-antibody and β-actin as a loading control (Figure 5A). When we investigated the surface expression levels of CD123 we found CD123 to be significantly downregulated in RhoH overexpressing cells (50%). Again, this could be correlated with significant changes in *IRF-*1 gene expression (Figure 5C) which showed a 15-fold downregulation of RhoH overexpressing cells compared to THP-1 cells. Therefore THP-1 cells show a phenotype similar to the one observed in siRhoH BaF3 cells, with low RhoH levels and upregulated *IRF-1 *and CD123 expression (5B and C).

## Discussion

Previous work has shown that RhoH is a negative regulator for growth, survival and cytoskeletal modifications [14]. We show here that the expression level of RhoH modulates the activity of STAT transcription factors STAT5 and STAT1. In the IL3-dependent cell line BaF3, RhoH acts as a specific negative regulator of IL3, but not Epo-induced proliferation and silencing of *RhoH *gene expression allows the cells to proliferate faster in response to IL3.

The JAK-STAT pathway is a major signalling pathway of haematopoietic cells that links proliferative signals to the cell cycle machinery. In IL3-mediated signalling, STAT5 plays a major role in the regulation of proliferation, differentiation and anti-apoptotic signalling [25,26]. We demonstrate that overexpression of RhoH leads to a decrease in the activity of STAT5, whereas silencing of RhoH expression causes an increased activity of STAT5 compared to control cells. No changes in the expression level of total STAT5 protein were detectable and we therefore conclude that RhoH does not modulate STAT5 activity through regulation of STAT5 expression levels. Most interestingly, we also could show a link between RhoH expression levels and changes in the surface expression of the ILR3 α-chain CD123.

It had previously been suggested that an elevated CD123 expression, as it can be found in patients with acute myeloid leukaemia (AML), may contribute to the increased proliferation of leukaemic blasts, hyperactivation of STAT5 and poor prognosis [28]. Low expression levels of RhoH were recently described as yet another factor linked to poor patient prognosis [13]. Our data now show that these two findings indeed might be connected. Because low RhoH expression leads to an increased STAT5 activity, STAT5 might then induce expression of the *IRF-1 *gene, which in turn allows an IRF-1-dependent upregulation of the *CD123 *gene, eventually leading to an increase in the surface levels of the protein.

Although the regulatory influence of RhoH on STAT5 activity would be sufficient to account for the differences in proliferation, we observed an additional mechanism by which RhoH negatively regulates IL3-induced growth, namely the activation of STAT1 in RhoH overexpressing cells. STAT1 is the key factor that transduces the antiproliferative effects of interferons [31] and activation of STAT1 coincides with cell cycle arrest or apoptosis. As a consequence, STAT1 knock-out mice develop tumours more rapidly [32,33]. When we screened control cells and RhoH overexpressing cells for differences in their sensitivity towards apoptotic stimuli, we were not able to find any. However, siRhoH showed increased survival after cytokine deprivation and readdition of IL3 to starved cells induced a pool of rapidly growing cells, whereas parental cells did not recover (unpublished data).

It has been reported that STAT1 activation can lead to the upregulation of *p21^Cip1 ^*causing subsequent cell cycle arrest or apoptosis and a STAT1 DNA binding site was found in the *p21^Cip1 ^*promoter [24]. Another member of this family, *p27^Kip1^*, was shown to be downregulated by IL3 and BCR-ABL [34]. Interestingly, we found that *p21^Cip1 ^*and *p27^Kip1 ^*are both upregulated when RhoH is expressed, i.e. STAT1 is activated, and we suggest this as a RhoH-dependent mechanism that serves to regulate progression in the cell cycle. We propose a model, where the balance between proliferation and apoptosis is fine-tuned by the expression level of RhoH. While high levels of RhoH lead to increased STAT1 but reduced STAT5 activity, downregulation of RhoH expression activates STAT5-dependent proliferation and survival signals. It will be important to examine whether in IL3 sensitive, differentiating haematopoietic progenitor cells, the expression level of RhoH can regulate the balance between proliferation and cell cycle arrest or apoptosis. There was no obvious haematopoietic defect in RhoH-deficient animals, however, it is possible that the disturbed IL3-dependent signalling can be compensated by other cytokines. In addition, it is known that in B cells, RhoH is a target of somatic hypermutation and translocation which affects the expression of the protein [10]. Nevertheless, RhoH-deficient animals did not develop lymphomas or show other B cell malignancies, which is a discrepancy that shows the limit of the animal model.

Two recent publications now link low RhoH protein levels to cancer [12,13]. In AML, RhoH expression is low, causing high levels of active, GTP-bound Rac1 and eventually resistance to chemotherapeutic apoptosis [13]. Our results indicate that other signalling pathways, such as STAT5 activation and high expression of the IL3-binding α chain, might additionally be modulated by RhoH and contribute to the disease. To understand the importance of RhoH for the development of haematopoietic malignancies, it will be crucial to establish a link between *RhoH *mutations, its expression on the protein level and the activity of signalling molecules such as STATs that are known to be upregulated in a number of myeloproliferative disorders [35,36]. In addition, the JAK-STAT pathway plays a central role in cytokine-mediated signalling in haematopoiesis and the immune system. This pathway has not yet been discussed as a potential target of RhoH and it will therefore be interesting to see whether cytokine receptors other than IL3 are regulated through the expression level of RhoH.

## Conclusions

Taken together, we show that the haematopoietic GTPase RhoH can modulate signalling through the JAK-STAT pathway. High levels of RhoH lead to preferential activation of STAT1 and reduced cell proliferation. While there were no changes in apoptosis detectable at high RhoH levels, we found a pronounced upregulation of *p21^Cip1 ^*and *p27^Kip^*, two genes involved in cell cycle arrest. Low RhoH levels led to an upregulation of IL3-dependent cell growth, STAT5 activity and an upregulation of CD123 surface expression. This phenotype was also found in human monocytic THP-1 cells, suggesting that a correction of low RhoH expression levels might be beneficial for AML patients.

## Methods

### Materials

Stimulation with IL3 was performed with recombinant IL3 (Natutec, Frankfurt, Germany). BaF3 cells were obtained from DSMZ (Braunschweig, Germany). All data shown were performed at least in three independent experiments.

### cDNAs cloning and sequencing

The puromycin resistance cassette was amplified by PCR from the vector pSilencer 5.1 U6 retro (Ambion, Austin, USA) and restriction sites for Sal-I and Xho-I were introduced. Sense primer puromycin resistance: 5'-GCT AAC GTC GAC CGG GTA GGG GAG GCG CT-3'; anti sense primer puromycin resistance: 5'-GCT AAT CTC GAG TCA GGC ACC GGG CTT GC-3'. The PCR product was cloned into the Sal-I restriction site of the pMX-IRES-CD4 vector [23]. The resulting construct (pMX-IRES-CD4-Puro) was verified by sequence analysis. Full length murine RhoH was cloned into BamH-I and Not-I sites of the pMX-IRES-CD4-Puro vector. The resulting construct pMX-RhoH-IRES-CD4-Puro RhoH was verified by sequence analysis.

### Cell culture reagents

BaF3 cells were maintained in RPMI1640 medium containing 10% FBS, 1% Pen/Strep and IL3 containing supernatant (0.5%) generated by the cell line X63Ag8-653 [37]. THP-1 cells were cultivated in RPMI1640 medium containing 10% FBS and 1% Pen/Strep.

### Retroviral vector transduction

Retroviral supernatants were generated and used to transduce the IL3-dependent pro-B cell line BaF3 as described [38]. Briefly, six-well plates of 293-derived Phoenix eco cells were transiently transfected with cDNAs encoding for murine RhoH gene (pMX-IRES-CD4-Puro) or the empty vector (control). After 48 h, 750 μl of viral supernatant was added to 5×10^5 ^BaF3 cells and centrifuged for 120 min at 37 °C and 900 g in the presence of 16 μg of Polybrene (Sigma, Taufkirchen, Germany). Transduced cells were selected in the presence of 1.5 μg/ml puromycin and transfection efficiency was evaluated by FACS analysis of human CD4 expression (BD Biosciences, Germany). To generate siRNAs specific to mouse RhoH a silencing 21mer as described in [14] was cloned into the vector pSilencer 5.1 U6 (Ambion, Austin, USA). As a control, a scrambled sequence with no similarity to a mouse gene was used. After infection, transduced cells were selected in the presence of 1.5 μg/ml puromycin.

### Transient transfections

THP-1 cells were transiently transfected with the human HA-tagged RhoH cDNA containing vector pMX-IRES-GFP or the corresponding empty vector using Metafectene (Biontex, Martinsried, Germany) according to manufacturer's instructions.

### FACS analysis

For the intracellular analysis of phosphorylated STATs, cells were fixed with 4% PFA/PBS prior to overnight permeabilization with methanol. Phosphorylated STATs were detected using FITC-labelled pSTAT1 (Y701), pSTAT5 (Y694) antibodies or the respective isotype controls (BD Bioscience, Heidelberg, Germany). For the analysis of CD123 surface expression, cells were incubated for 45 min with PBS/2% FBS before labelling with murine CD123-PE or human CD123-APC antibodies (eBioscience, Frankfurt a.M, Germany), or the respective isotype controls (BD Biosciences, Heidelberg, Germany). Cells were analyzed on a FACS Canto (BD Biosciences, Heidelberg, Germany).

### Measurement of Cell Viability

Cytokine-dependent growth was determined by quantification of cellular ATP using the Cell Titer Glo Assay (Promega, Madison, WI, USA). Cells were washed with RPMI and starved for 3 hours in the presence of 1 mg/ml BSA. 3.75 × 10^4 ^cells/ml were seeded in a 96 well plate with the corresponding cytokine concentrations. Cells were processed according to the manufacturer's protocol and luminescence was determined using a Lumistar Optima luminometer (BMG Labtech, Offenburg, Germany).

### Annexin V Assay

Cells were depleted of IL3 for 3 hours and 2.5 × 10^5 ^BaF3 cells/ml were seeded in a 6-well plate. Cells were either incubated overnight in regular BaF3 cell medium, in the absence of IL3 or under other stress conditions, such as treatment with 1 μM doxorubicin or 1 μM staurosporine (Sigma, Taufkirchen, Germany). Cells were stained with Annexin-V and propidium iodide according to the Annexin-V-FLUOS kit protocol provided by the manufacturer (Roche, Penzberg, Germany). Apoptosis was quantified using a FACS Canto (BD Biosciences, Heidelberg, Germany).

### Whole Cell Extracts and Immunoprecipitation

BaF3 cells were starved for 3 h without IL3 and FBS before stimulation of 1 × 10^7 ^cells with 50 ng/ml IL3. Cells were lysed in NP40 lysis buffer with protease and phosphatase inhibitors (Roche, Penzberg, Germany) and incubated for 45 min at 4°C. After centrifugation, lysates were immunoprecipitated overnight with 5 μl of STAT1 or STAT5 antibodies (Santa Cruz Biotechnology, Santa Cruz, USA) bound to Protein A/G Sepharose (Santa Cruz Biotechnology, Santa Cruz, USA). Samples were separated by 12% SDS-PAGE, transferred to nitrocellulose and incubated with the corresponding phospho-specific antibodies for STAT1 (701) or STAT5 (Y694) (Cell Signaling Technology, Danvers MA, USA) or total STAT1/STAT5, followed by incubation with HRP-coupled anti-rabbit antibody (Cell Signaling Technology, Danvers MA, USA) and detection by enhanced chemiluminescence.

Detection of proteins after western blotting of whole cell lysates (20 μg protein) was performed using antibodies directed against β-actin and p27*^Kip1 ^*(Cell Signaling Technology, Danvers MA, USA), p21*^Cip1^*, STAT5 or HA-tag (Santa Cruz Biotechnology, Santa Cruz, USA). Quantification of immunoblots was performed using the Image Analysis System Bioprofil (Fröbel, Germany) Bio ID software version v12.06. Signal intensity was calculated against the loading control and is presented as fold induction compared with the unstimulated control or cells transduced with the empty vector (mean ± SD; *n *≥ 2). Statistical significance was assessed by using a paired 's *t*-test, with *P < 0.05, **P < 0.01 and ***P < 0.005.

### Quantitative real-time PCR

Small-scale preparations of RNA were made from 1 × 10^6 ^cells using the High Pure RNA Isolation Kit (Roche, Penzberg, Germany). Total RNA was transcribed with First Strand cDNA Kit (Fermentas GmbH, St. Leon-Rot, Germany). Aliquots of the cDNA were used for quantitative PCR analysis using the 7900 HT Fast Real-Time PCR System (Applied Biosystems, Darmstadt, Germany) and the ABsolute QPCR SYBR Green Rox Mix (Abgene, Epson, UK). The following primers were used: murine gapdh sense 5'-TTC ACC ACC ATG GAG AAG GC-3' and antisense 5'-GGC ATG GAC TGT GGT CAT GA-3', human gapdh sense 5'-ACG GAT TTG GTC GTA TTG GGC-3' and antisense 5'-TTG ACG GTG CCA TGG AAT TTG-3', murine RhoH sense 5'-GCT ACT CTG TGG CCA ACC AT-3' and antisense 5'-AGG TCC CAC CTC TCT CTG GT-3', p27 sense 5'-AGG GCC AAC AGA ACA GAA GA-3' and antisense 5'-CTC CTG GCA GGC AAC TAA TC-3'and p21 sense 5'-GCA GAC CAG CCT GAC-3' and antisense 5'-GCA GGC AGC GTA TAT ACA GGA-3', murine IRF-1 sense 5'-GGA GAT GTT AGC CGG ACA CT-3', murine IRF-1 antisense 5'-TGC TGA CGA CAC ACG GTG A-3', human IRF-1 sense 5'-GCT GGA CAT CAA CAA AGG AT-3' and antisense 5'-TGG TCT TTC ACC TCC TCG AT-3'. Results were analyzed using the Abgene software. For further analysis, results were exported to Excel (Microsoft) and calculated by relative ddCt method. All results were normalised with respect to the reference gene *GAPDH*. Results were then normalised to control cells (mean ± SD; *n≥*2). Statistical significance was assessed using a paired 's *t*-test, with *P < 0.05, **P < 0.01 or with ***P < 0.005.

## Abbreviations

AML: acute myeloid leukaemia; BMC: bone marrow cells; CD: cluster of differentiation; CDKI: cyclin dependent kinase inhibitors; GM-CSF: granulocyte macrophage-colony stimulating factor; HCL: hairy cell leukaemia; IL3: interleukin 3; IRF: interferon regulatory factor; ITAM: immune receptor tyrosine based activation motif; JAK: Janus kinase; RLU: relative light units; STAT: signal transducer and activator of transcription

## Competing interests

The authors declare that they have no competing interests.

## Authors' contributions

MSG and HL carried out cell culture and biochemical experiments, DM and DH performed FACS experiments, MA participated in the design of experiments using THP-1 cells, KA and KH participated in the coordination of the experiments and helped to draft the manuscript and KFK designed the experiments and wrote the manuscript. All authors read and approved the final manuscript.

## References

[B1] Madaule P Axel R A novel ras-related gene family Cell 1985 41 31 40 10.1016/0092-8674(85)90058-3 3888408

[B2] Bar-Sagi D Hall A Ras and Rho GTPases: a family reunion Cell 2000 103 227 238 10.1016/S0092-8674(00)00115-X 11057896

[B3] Vojtek AB Der CJ Increasing complexity of the Ras signaling pathway J Biol Chem 1998 273 19925 19928 10.1074/jbc.273.32.19925 9685325

[B4] Etienne-Manneville S Hall A Rho GTPases in cell biology Nature 2002 420 629 635 10.1038/nature01148 12478284

[B5] Dallery E Galiegue-Zouitina S Collyn-d'Hooghe M Quief S Denis C Hildebrand MP Lantoine D Deweindt C Tilly H Bastard C TTF, a gene encoding a novel small G protein, fuses to the lymphoma-associated LAZ3 gene by t(3;4) chromosomal translocation Oncogene 1995 10 2171 2178 7784061

[B6] Didsbury J Weber RF Bokoch GM Evans T Snyderman R rac, a novel ras-related family of proteins that are botulinum toxin substrates J Biol Chem 1989 264 16378 16382 2674130

[B7] Li X Bu X Lu B Avraham H Flavell RA Lim B The hematopoiesis-specific GTP-binding protein RhoH is GTPase deficient and modulates activities of other Rho GTPases by an inhibitory function Mol Cell Biol 2002 22 1158 1171 10.1128/MCB.22.4.1158-1171.2002 11809807 PMC134637

[B8] Zohn IM Campbell SL Khosravi-Far R Rossman KL Der CJ Rho family proteins and Ras transformation: the RHOad less traveled gets congested Oncogene 1998 17 1415 1438 10.1038/sj.onc.1202181 9779988

[B9] Gu Y Chae HD Siefring JE Jasti AC Hildeman DA Williams DA RhoH GTPase recruits and activates Zap70 required for T cell receptor signaling and thymocyte development Nat Immunol 2006 7 1182 1190 10.1038/ni1396 17028588

[B10] Fueller F Kubatzky KF The small GTPase RhoH is an atypical regulator of haematopoietic cells Cell Commun Signal 2008 6 6 10.1186/1478-811X-6-6 18823547 PMC2565660

[B11] Pasqualucci L Neumeister P Goossens T Nanjangud G Chaganti RS Kuppers R Dalla-Favera R Hypermutation of multiple proto-oncogenes in B-cell diffuse large-cell lymphomas Nature 2001 412 341 346 10.1038/35085588 11460166

[B12] Galiegue-Zouitina S Delestre L Dupont C Troussard X Shelley CS Underexpression of RhoH in Hairy Cell Leukemia Cancer Res 2008 68 4531 4540 10.1158/0008-5472.CAN-07-5661 18559497

[B13] Iwasaki T Katsumi A Kiyoi H Tanizaki R Ishikawa Y Ozeki K Kobayashi M Abe A Matsushita T Watanabe T Prognostic implication and biological roles of RhoH in acute myeloid leukaemia Eur J Haematol 2008 81 454 460 10.1111/j.1600-0609.2008.01132.x 18691253

[B14] Gu Y Jasti AC Jansen M Siefring JE RhoH, a hematopoietic-specific Rho GTPase, regulates proliferation, survival, migration, and engraftment of hematopoietic progenitor cells Blood 2005 105 1467 1475 10.1182/blood-2004-04-1604 15494435

[B15] Chae HD Lee KE Williams DA Gu Y Cross-talk between RhoH and Rac1 in regulation of actin cytoskeleton and chemotaxis of hematopoietic progenitor cells Blood 2008 111 2597 2605 10.1182/blood-2007-06-093237 18089848 PMC2254535

[B16] Dorn T Kuhn U Bungartz G Stiller S Bauer M Ellwart J Peters T Scharffetter-Kochanek K Semmrich M Laschinger M RhoH is important for positive thymocyte selection and T-cell receptor signaling Blood 2007 109 2346 2355 10.1182/blood-2006-04-019034 17119112

[B17] Daryadel A Yousefi S Troi D Schmid I Schmidt-Mende J Mordasini C Dahinden CA Ziemiecki A Simon HU RhoH/TTF negatively regulates leukotriene production in neutrophils J Immunol 2009 182 6527 6532 10.4049/jimmunol.0803846 19414807

[B18] Oda H Fujimoto M Patrick MS Chida D Sato Y Azuma Y Aoki H Abe T Suzuki H Shirai M RhoH plays critical roles in Fc epsilon RI-dependent signal transduction in mast cells J Immunol 2009 182 957 962 19124738 10.4049/jimmunol.182.2.957

[B19] Yousefi S Cooper PR Mueck B Potter SL Jarai G cDNA representational difference analysis of human neutrophils stimulated by GM-CSF Biochem Biophys Res Commun 2000 277 401 409 10.1006/bbrc.2000.3678 11032736

[B20] Migliaccio G Migliaccio AR Visser JW Synergism between erythropoietin and interleukin-3 in the induction of hematopoietic stem cell proliferation and erythroid burst colony formation Blood 1988 72 944 951 3262001

[B21] Lantz CS Boesiger J Song CH Mach N Kobayashi T Mulligan RC Nawa Y Dranoff G Galli SJ Role for interleukin-3 in mast-cell and basophil development and in immunity to parasites Nature 1998 392 90 93 10.1038/32190 9510253

[B22] Rodewald HR Dessing M Dvorak AM Galli SJ Identification of a committed precursor for the mast cell lineage Science 1996 271 818 822 10.1126/science.271.5250.818 8629001

[B23] Liu X Constantinescu SN Sun Y Bogan JS Hirsch D Weinberg RA Lodish HF Generation of mammalian cells stably expressing multiple genes at predetermined levels Anal Biochem 2000 280 20 28 10.1006/abio.2000.4478 10805516

[B24] Chin YE Kitagawa M Su WC You ZH Iwamoto Y Fu XY Cell growth arrest and induction of cyclin-dependent kinase inhibitor p21 WAF1/CIP1 mediated by STAT1 Science 1996 272 719 722 10.1126/science.272.5262.719 8614832

[B25] Mui AL Wakao H O'Farrell AM Harada N Miyajima A Interleukin-3, granulocyte-macrophage colony stimulating factor and interleukin-5 transduce signals through two STAT5 homologs EMBO J 1995 14 1166 1175 7720707 10.1002/j.1460-2075.1995.tb07100.x PMC398194

[B26] Quelle FW Sato N Witthuhn BA Inhorn RC Eder M Miyajima A Griffin JD Ihle JN JAK2 associates with the beta c chain of the receptor for granulocyte-macrophage colony-stimulating factor, and its activation requires the membrane-proximal region Mol Cell Biol 1994 14 4335 4341 8007942 10.1128/mcb.14.7.4335-4341.1994 PMC358804

[B27] Mui AL Wakao H Kinoshita T Kitamura T Miyajima A Suppression of interleukin-3-induced gene expression by a C-terminal truncated Stat5: role of Stat5 in proliferation EMBO J 1996 15 2425 2433 8665850 PMC450174

[B28] Testa U Riccioni R Militi S Coccia E Stellacci E Samoggia P Latagliata R Mariani G Rossini A Battistini A Elevated expression of IL-3Ralpha in acute myelogenous leukemia is associated with enhanced blast proliferation, increased cellularity, and poor prognosis Blood 2002 100 2980 2988 10.1182/blood-2002-03-0852 12351411

[B29] Guzman ML Upchurch D Grimes B Howard DS Rizzieri DA Luger SM Phillips GL Jordan CT Expression of tumor-suppressor genes interferon regulatory factor 1 and death-associated protein kinase in primitive acute myelogenous leukemia cells Blood 2001 97 2177 2179 10.1182/blood.V97.7.2177 11264190

[B30] Tian SS Tapley P Sincich C Stein RB Rosen J Lamb P Multiple signaling pathways induced by granulocyte colony-stimulating factor involving activation of JAKs, STAT5, and/or STAT3 are required for regulation of three distinct classes of immediate early genes Blood 1996 88 4435 4444 8977235

[B31] Bromberg JF Horvath CM Wen Z Schreiber RD Darnell JE Transcriptionally active Stat1 is required for the antiproliferative effects of both interferon alpha and interferon gamma Proc Natl Acad Sci USA 1996 93 7673 7678 10.1073/pnas.93.15.7673 8755534 PMC38805

[B32] Durbin JE Hackenmiller R Simon MC Levy DE Targeted disruption of the mouse Stat1 gene results in compromised innate immunity to viral disease Cell 1996 84 443 450 10.1016/S0092-8674(00)81289-1 8608598

[B33] Meraz MA White JM Sheehan KC Bach EA Rodig SJ Dighe AS Kaplan DH Riley JK Greenlund AC Campbell D Targeted disruption of the Stat1 gene in mice reveals unexpected physiologic specificity in the JAK-STAT signaling pathway Cell 1996 84 431 442 10.1016/S0092-8674(00)81288-X 8608597

[B34] Parada Y Banerji L Glassford J Lea NC Collado M Rivas C Lewis JL Gordon MY Thomas NS Lam EW BCR-ABL and interleukin 3 promote haematopoietic cell proliferation and survival through modulation of cyclin D2 and p27Kip1 expression J Biol Chem 2001 276 23572 23580 10.1074/jbc.M101885200 11323429

[B35] Akala OO Clarke MF Hematopoietic stem cell self-renewal Curr Opin Genet Dev 2006 16 496 501 10.1016/j.gde.2006.08.011 16919448

[B36] Lin TS Mahajan S Frank DA STAT signaling in the pathogenesis and treatment of leukemias Oncogene 2000 19 2496 2504 10.1038/sj.onc.1203486 10851048

[B37] Karasuyama H Melchers F Establishment of mouse cell lines which constitutively secrete large quantities of interleukin 2, 3, 4 or 5, using modified cDNA expression vectors Eur J Immunol 1988 18 97 104 10.1002/eji.1830180115 2831066

[B38] Kubatzky KF Liu W Goldgraben K Simmerling C Smith SO Constantinescu SN Structural requirements of the extracellular to transmembrane domain junction for erythropoietin receptor function J Biol Chem 2005 280 14844 14854 10.1074/jbc.M411251200 15657048

